# Crystal structure of an Ag^I^ inter­calation compound: *catena*-poly[[silver(I)-μ-*N*-(pyridin-3-ylmeth­yl)pyridin-3-amine-κ^2^ *N*:*N*′] hexa­fluorido­phosphate aceto­nitrile disolvate]

**DOI:** 10.1107/S2056989017013421

**Published:** 2017-09-25

**Authors:** Suk-Hee Moon, Youngjin Kang, Ki-Min Park

**Affiliations:** a Department of Food and Nutrition, Kyungnam College of Information and Technology, Busan 47011, Republic of Korea; b Division of Science Education, Kangwon National University, Chuncheon 24341, Republic of Korea; c Research institute of Natural Science, Gyeongsang National University, Jinju 52828, Republic of Korea

**Keywords:** crystal structure, silver(I), dipyridyl-type ligand, inter­calation, Ag⋯Ag inter­action, hydrogen bonding, π–π inter­actions

## Abstract

The asymmetric unit in the title compound, [Ag(C_11_H_11_N_3_)]PF_6_·2CH_3_CN or {[Ag*L*]·PF_6_·2CH_3_CN}_*n*_, *L* = *N*-(pyridin-3-ylmeth­yl)pyridin-3-amine, comprises one Ag^I^ atom, one *L* ligand, two aceto­nitrile solvent mol­ecules and one PF_6_^−^ anion disordered over two orientations in a 0.567 (11):0.433 (11) ratio. Each Ag^I^ atom is coordinated by two pyridine N atoms from two *L* ligands in a slightly distorted linear coordination geometry [N—Ag—N = 170.55 (8)°]. Each *L* ligand bridges two Ag^I^ ions, resulting in the formation of a zigzag chain propagating along the [101] direction. In the crystal, Ag⋯Ag contacts [3.3023 (5) Å] and inter­molecular π–π stacking inter­actions [centroid-to-centroid distance = 3.5922 (15) Å] between the pyridine rings link these chains into a corrugated layer parallel to the (01) plane. The layers are stacked with a separation of 10.4532 (5) Å, and aceto­nitrile solvent mol­ecules and PF_6_^−^ anions as guests are inter­calated between the layers. The layers are connected through several N/C—H⋯F hydrogen bonds and P—F⋯π inter­actions [F⋯ring centroid = 3.241 (8) Å] between the layer and the inter­calated guests and between the inter­calated guests, forming a three-dimensional supra­molecular network.

## Abstract 2

The Ag^I^ atom in the title compound is two-coordinate, being bound to two pyridine N atoms from two *N*-(pyridin-3-ylmeth­yl)pyridin-3-amine ligands in a slightly distorted linear fashion. Each Ag^I^ ion bridges the dipyridyl-type ligands to form polymeric zigzag chains. The chains are connected *via* Ag⋯Ag and π–π inter­actions, forming a corrugated layer parallel to (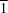
01). Aceto­nitrile solvent mol­ecules and PF_6_^−^ anions are inter­calated between these layers. Several inter­molecular N/C—H⋯F hydrogen bonds lead to formation of a three-dimensional supra­molecular network.

## Chemical context   

Silver coordination polymers based on dipyridyl-type ligands have been widely exploited due to the intriguing topologies and the fascinating properties caused by a variety of coordination geometries and *d* ^10^ electronic configurations of the Ag^I^ ion (Leong & Vittal, 2011 ▸; Moulton & Zaworotko, 2001 ▸; Wang *et al.*, 2012 ▸). In particular, Ag^I^ ions have a preference for a linear two-coordinate geometry and can serve to link bridging dipyridyl-type ligands to form polymeric chains. Based on this concept, we have focused our attention on the development of one-dimensional Ag^I^ coordination polymers with dipyridyl-type ligands. Up to date, we have reported several Ag^I^ coord­ination polymers with inter­esting topologies involving zigzag (Moon *et al.*, 2016 ▸), helical (Moon *et al.*, 2014 ▸, 2015 ▸) and double helical (Lee *et al.*, 2015 ▸) structures. In an extension of our research, the title compound was prepared by the reaction of silver(I) hexa­fluorido­phosphate with a dipyridyl type-ligand, namely *N*-(pyridin-3-ylmeth­yl)pyridin-3-amine (*L*), synthesized according to a literature procedure (Lee *et al.*, 2013 ▸). Herein, we report on the crystal structure of the title compound in which lattice solvent mol­ecules and anions as guests are inter­calated between the layers formed by inter­molecular inter­actions between zigzag –(Ag–*L*)_*n*_– chains.
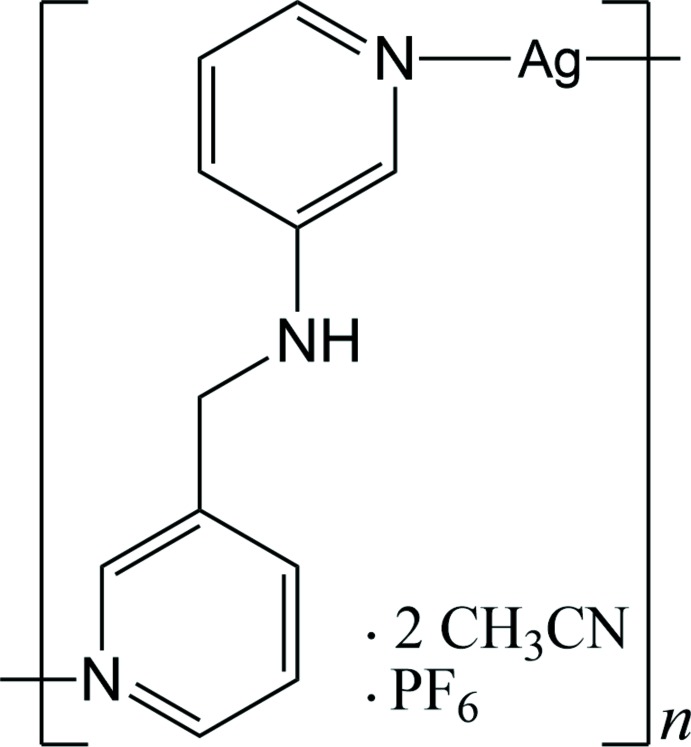


## Structural commentary   

**Figure 1:**
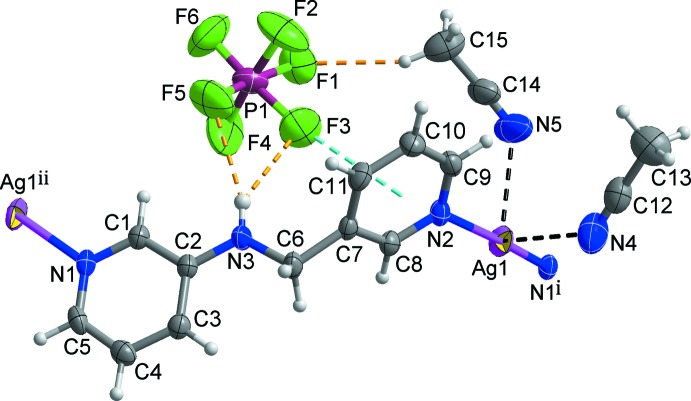
View of the mol­ecular structure of the title compound, showing the atom-numbering scheme. Displacement ellipsoids are drawn at the 50% probability level. Disordered F atoms of the PF_6_^−^ anion have been omitted for clarity. Black and yellow dashed lines represent Ag⋯N inter­actions and inter­molecular C/N—H⋯F hydrogen bonds, respectively. [Symmetry codes: (i) *x* + 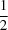, −*y* + 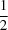, *z* + 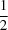; (ii) *x* − 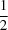, −*y* + 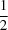, *z* − 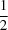.]

The mol­ecular components of the title structure are shown in Fig. 1 ▸. The asymmetric unit comprises one Ag^I^ atom, one *L* ligand, two aceto­nitrile solvent mol­ecules, and one hexa­fluorido­phosphate anion disordered over two orientations in a 0.567 (11):0.433 (11) ratio. The silver(I) atom is coordinated by two pyridine N atoms (N1 and N2) from two symmetry-related *L* ligands, leading to the formation of an infinite zigzag chain propagating along the [101] direction. Thus, the Ag^I^ atom is two-coordinated in a slightly distorted linear coordination geometry [N1^i^—Ag1—N2 = 170.55 (8)°; symmetry code: (i) *x* + 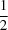, −*y* + 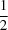, *z* + 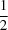; Table 1 ▸]. This distortion from linear geometry may be caused by Ag⋯N inter­actions between the Ag^I^ ion and two aceto­nitrile N atoms [Ag1⋯N4 = 2.792 (4), Ag1⋯N5 = 2.815 (4) Å; black dashed lines in Fig. 1 ▸]. The two pyridine rings coordinated to the Ag^I^ center are tilted slightly, by 6.29 (15)° with respect to each other. In the chain, the Ag^I^ atoms are separated by 11.1009 (3) Å along the *L* linker which adopts a stretched *trans* conformation with the C2—N3—C6—C7 torsion angles being 174.7 (3)°.

## Supra­molecular features   

**Figure 2:**
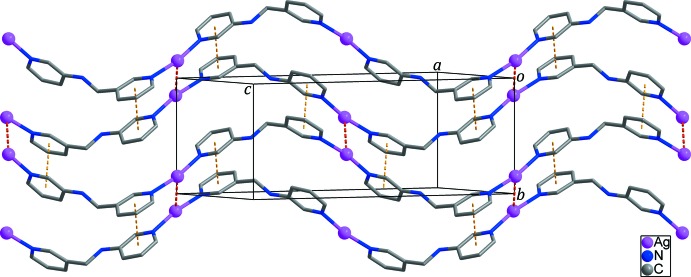
The two-dimensional network formed through Ag⋯Ag contacts (red dashed lines) and inter­molecular π–π stacking inter­actions (yellow dashed lines). Aceto­nitrile solvent mol­ecules, the PF_6_^−^ anions and H atoms have been omitted for clarity.

**Figure 3:**
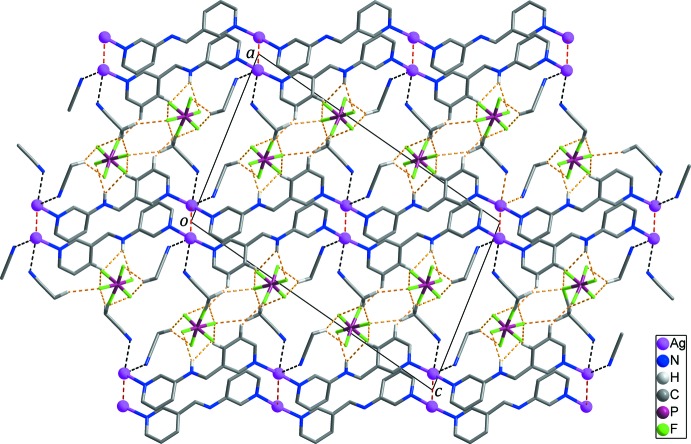
Inter­layer stacking showing the inter­calation of aceto­nitrile mol­ecules and PF_6_^−^ anions between the layers. Red, black and yellow dashed lines represent Ag⋯Ag contacts, Ag⋯N inter­actions and N/C—H⋯F hydrogen bonds, respectively. Disordered F atoms of the PF_6_^−^ anions and H atoms not involved in inter­molecular inter­actions have been omitted for clarity.

The neighbouring zigzag chains are connected by Ag⋯Ag contacts [Ag1⋯Ag1 = 3.3023 (5) Å; red dashed lines in Fig. 2 ▸] and inter­molecular π-π-stacking inter­actions between the pyridine rings [*Cg*1⋯*Cg*2^ii^ = 3.5922 (15) Å; yellow dashed lines in Fig. 2 ▸; *Cg*1 and *Cg*2 are the centroids of the N1/C1–C5 and N2/C7–C11 rings, respectively; symmetry code: (ii) −*x* + 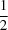, *y* − 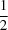, −*z* + 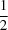], resulting in the formation of a corrugated layer spreading out along the (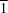
01) plane (Fig. 2 ▸). Adjacent layers are stacked on each other with a separation of 10.4532 (5) Å. Aceto­nitrile mol­ecules and PF_6_^−^ anions as guests are inter­calated between the layers (Fig. 3 ▸). The layers are further connected by several inter­molecular N/C—H⋯F hydrogen bonds (Table 2 ▸; yellow dashed lines in Figs. 1 ▸ and 3 ▸) and P—F⋯π inter­actions [F3⋯*Cg*2 = 3.241 (8) Å; sky-blue dashed line in Fig. 1 ▸] between the layer and the anions and between the aceto­nitrile solvent mol­ecules and the anions, forming a three-dimensional supra­molecular network.

## Synthesis and crystallization   

The *L* ligand was synthesized according to a literature method (Lee *et al.*, 2013 ▸). Slow evaporation of an aceto­nitrile solution of the *L* ligand with AgPF_6_ in the molar ratio 1:1 afforded colourless block-like X-ray quality single crystals of the title compound.

## Refinement   

Crystal data, data collection and structure refinement details are summarized in Table 3 ▸. The PF_6_^−^ anion is disordered over two orientations in a 0.567 (11):0.433 (11) ratio. The amine H atom was located from a difference-Fourier map and freely refined [N—H = 0.84 (3) Å]. All other H atoms were positioned geometrically and refined as riding: C—H = 0.95 Å for C*sp* ^2^—H, 0.99 Å for methyl­ene C—H and 0.98 Å for methyl C—H with *U*_iso_(H) = 1.5*U*_eq_ (C-meth­yl) and 1.2*U*_eq_(C) for other C-bound H atoms.

## Supplementary Material

Crystal structure: contains datablock(s) I, New_Global_Publ_Block. DOI: 10.1107/S2056989017013421/su5393sup1.cif

Structure factors: contains datablock(s) I. DOI: 10.1107/S2056989017013421/su5393Isup2.hkl

CCDC reference: 1575393

Additional supporting information:  crystallographic information; 3D view; checkCIF report

## Figures and Tables

**Table 1:** Selected geometric parameters (Å, °)

Ag1—N1^i^	2.163 (2)	Ag1—N2	2.166 (2)
			
N1^i^—Ag1—N2	170.55 (8)		

Symmetry code: (i) 
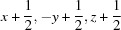
.

**Table 2:** Hydrogen-bond geometry (Å, °)

*D*—H⋯*A*	*D*—H	H⋯*A*	*D*⋯*A*	*D*—H⋯*A*
N3—H3*A*⋯F3	0.84 (3)	2.49 (3)	3.208 (8)	144 (3)
N3—H3*A*⋯F5	0.84 (3)	2.46 (3)	3.273 (10)	162 (3)
N3—H3*A*⋯F5′	0.84 (3)	2.18 (3)	2.999 (10)	167 (3)
C8—H8⋯N4^ii^	0.95	2.63	3.478 (5)	149
C10—H10⋯F2^iii^	0.95	2.39	3.176 (9)	140
C13—H13*A*⋯F2′^iii^	0.98	2.54	3.487 (13)	163
C13—H13*B*⋯F6^i^	0.98	2.53	3.365 (10)	144
C13—H13*B*⋯F1′^i^	0.98	2.59	3.57 (2)	171
C15—H15*A*⋯F1	0.98	2.52	3.441 (9)	157
C15—H15*A*⋯F3′	0.98	2.42	3.356 (13)	160
C15—H15*B*⋯F2^iv^	0.98	2.23	3.088 (10)	146
C15—H15*B*⋯F5′^iv^	0.98	2.57	3.509 (11)	160
C15—H15*C*⋯F1^iii^	0.98	2.36	3.329 (8)	168
C15—H15*C*⋯F1′^iii^	0.98	2.09	3.037 (9)	161

Symmetry codes: (i) 
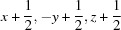
; (ii) 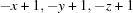; (iii) 
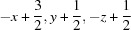
; (iv) 
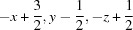
.

**Table 3:** Experimental details

Crystal data	
Chemical formula	[Ag(C_11_H_11_N_3_)]PF_6_·2C_2_H_3_N
*M*_r_	520.17
Crystal system, space group	Monoclinic, *P*2_1_/*n*
Temperature (K)	173
*a*, *b*, *c* (Å)	12.8997 (4), 7.5361 (3), 20.9747 (7)
β (°)	102.9900 (6)
*V* (Å^3^)	1986.84 (12)
*Z*	4
Radiation type	Mo *K*α
μ (mm^−1^)	1.16
Crystal size (mm)	0.35 × 0.25 × 0.15

Data collection	
Diffractometer	Bruker APEXII CCD
Absorption correction	Multi-scan (*SADABS*; Bruker, 2014 ▸)
*T*_min_, *T*_max_	0.666, 0.746
No. of measured, independent and observed [*I* > 2σ(*I*)] reflections	11824, 4316, 3527
*R*_int_	0.020
(sin θ/λ)_max_ (Å^−1^)	0.639

Refinement	
*R*[*F* ^2^ > 2σ(*F* ^2^)], *wR*(*F* ^2^), *S*	0.031, 0.081, 1.10
No. of reflections	4316
No. of parameters	312
No. of restraints	18
H-atom treatment	H atoms treated by a mixture of independent and constrained refinement
Δρ_max_, Δρ_min_ (e Å^−3^)	1.12, −0.66

Computer programs: *APEX2* and *SAINT* (Bruker, 2014 ▸), *SHELXS97* and *SHELXTL* (Sheldrick, 2008 ▸), *SHELXL2014* (Sheldrick, 2015 ▸), *DIAMOND* (Brandenburg, 2010 ▸) and *publCIF* (Westrip, 2010 ▸).

## supplementary crystallographic information

### Crystal data

**Table:**

[Ag(C_11_H_11_N_3_)]PF_6_·2C_2_H_3_N	*F*(000) = 1032
*M**_r_* = 520.17	*D*_x_ = 1.739 Mg m^−^^3^
Monoclinic, *P*2_1_/*n*	Mo *K*α radiation, λ = 0.71073 Å
*a* = 12.8997 (4) Å	Cell parameters from 5825 reflections
*b* = 7.5361 (3) Å	θ = 2.9–28.0°
*c* = 20.9747 (7) Å	µ = 1.16 mm^−^^1^
β = 102.9900 (6)°	*T* = 173 K
*V* = 1986.84 (12) Å^3^	Block, colorless
*Z* = 4	0.35 × 0.25 × 0.15 mm

### Data collection

**Table:**

Bruker APEXII CCD diffractometer	3527 reflections with *I* > 2σ(*I*)
φ and ω scans	*R*_int_ = 0.020
Absorption correction: multi-scan (SADABS; Bruker, 2014)	θ_max_ = 27.0°, θ_min_ = 1.7°
*T*_min_ = 0.666, *T*_max_ = 0.746	*h* = −10→16
11824 measured reflections	*k* = −9→9
4316 independent reflections	*l* = −26→26

### Refinement

**Table:**

Refinement on *F*^2^	Primary atom site location: structure-invariant direct methods
Least-squares matrix: full	Secondary atom site location: difference Fourier map
*R*[*F*^2^ > 2σ(*F*^2^)] = 0.031	Hydrogen site location: mixed
*wR*(*F*^2^) = 0.081	H atoms treated by a mixture of independent and constrained refinement
*S* = 1.10	*w* = 1/[σ^2^(*F*_o_^2^) + (0.0297*P*)^2^ + 2.2488*P*] where *P* = (*F*_o_^2^ + 2*F*_c_^2^)/3
4316 reflections	(Δ/σ)_max_ = 0.001
312 parameters	Δρ_max_ = 1.12 e Å^−^^3^
18 restraints	Δρ_min_ = −0.66 e Å^−^^3^

### Special details

**Table:**

Geometry. All esds (except the esd in the dihedral angle between two l.s. planes) are estimated using the full covariance matrix. The cell esds are taken into account individually in the estimation of esds in distances, angles and torsion angles; correlations between esds in cell parameters are only used when they are defined by crystal symmetry. An approximate (isotropic) treatment of cell esds is used for estimating esds involving l.s. planes.

### Fractional atomic coordinates and isotropic or equivalent isotropic displacement parameters (Å^2^)

**Table:**

	*x*	*y*	*z*	*U*_iso_*/*U*_eq_	Occ. (<1)
Ag1	0.57809 (2)	0.34509 (3)	0.48065 (2)	0.04382 (9)	
N1	0.09170 (18)	0.3315 (3)	0.06343 (10)	0.0313 (5)	
N2	0.55270 (18)	0.4849 (3)	0.38820 (10)	0.0317 (5)	
N3	0.3023 (2)	0.4904 (4)	0.19724 (12)	0.0385 (6)	
H3A	0.352 (3)	0.439 (4)	0.1850 (15)	0.035 (8)*	
C1	0.1875 (2)	0.3614 (4)	0.10237 (12)	0.0305 (6)	
H1	0.2478	0.3053	0.0924	0.037*	
C2	0.2022 (2)	0.4724 (3)	0.15729 (12)	0.0282 (5)	
C3	0.1127 (2)	0.5557 (4)	0.17056 (13)	0.0315 (6)	
H3	0.1192	0.6331	0.2070	0.038*	
C4	0.0141 (2)	0.5233 (4)	0.12960 (13)	0.0347 (6)	
H4	−0.0477	0.5784	0.1379	0.042*	
C5	0.0059 (2)	0.4111 (4)	0.07669 (13)	0.0328 (6)	
H5	−0.0620	0.3897	0.0490	0.039*	
C6	0.3223 (2)	0.6106 (4)	0.25170 (13)	0.0363 (6)	
H6A	0.2721	0.5853	0.2800	0.044*	
H6B	0.3097	0.7338	0.2354	0.044*	
C7	0.4343 (2)	0.5940 (3)	0.29117 (12)	0.0288 (5)	
C8	0.4549 (2)	0.5027 (3)	0.34988 (12)	0.0294 (5)	
H8	0.3968	0.4500	0.3638	0.035*	
C9	0.6343 (2)	0.5596 (4)	0.36808 (14)	0.0355 (6)	
H9	0.7041	0.5474	0.3946	0.043*	
C10	0.6207 (2)	0.6531 (4)	0.31048 (14)	0.0400 (7)	
H10	0.6801	0.7041	0.2976	0.048*	
C11	0.5193 (2)	0.6720 (4)	0.27147 (13)	0.0374 (6)	
H11	0.5082	0.7374	0.2318	0.045*	
P1	0.52792 (8)	0.16310 (12)	0.15620 (5)	0.0532 (2)	
F1	0.6049 (5)	0.0241 (8)	0.2082 (3)	0.0812 (19)	0.567 (11)
F2	0.6355 (6)	0.2633 (12)	0.1590 (6)	0.106 (3)	0.567 (11)
F3	0.5178 (6)	0.2625 (10)	0.2228 (4)	0.106 (3)	0.567 (11)
F4	0.4273 (13)	0.060 (3)	0.1615 (8)	0.133 (6)	0.567 (11)
F5	0.4615 (6)	0.3036 (14)	0.1156 (4)	0.100 (3)	0.567 (11)
F6	0.5453 (8)	0.0508 (11)	0.0998 (4)	0.119 (3)	0.567 (11)
F1'	0.5583 (12)	−0.0286 (12)	0.1587 (9)	0.149 (7)	0.433 (11)
F2'	0.6253 (9)	0.2192 (19)	0.1252 (6)	0.106 (4)	0.433 (11)
F3'	0.5773 (14)	0.211 (2)	0.2223 (5)	0.184 (7)	0.433 (11)
F4'	0.4247 (14)	0.101 (3)	0.1739 (9)	0.128 (7)	0.433 (11)
F5'	0.4867 (8)	0.3671 (11)	0.1435 (6)	0.086 (3)	0.433 (11)
F6'	0.4650 (11)	0.1392 (17)	0.0770 (4)	0.125 (5)	0.433 (11)
N4	0.6922 (3)	0.6394 (5)	0.53962 (16)	0.0733 (10)	
C12	0.7722 (3)	0.6996 (5)	0.53960 (16)	0.0538 (9)	
C13	0.8750 (3)	0.7783 (6)	0.5397 (2)	0.0756 (12)	
H13A	0.8918	0.7604	0.4969	0.113*	
H13B	0.9298	0.7219	0.5736	0.113*	
H13C	0.8727	0.9057	0.5487	0.113*	
N5	0.7553 (3)	0.1622 (7)	0.45595 (18)	0.0922 (14)	
C14	0.7749 (3)	0.1581 (5)	0.40711 (19)	0.0566 (9)	
C15	0.7958 (4)	0.1556 (6)	0.3423 (2)	0.0749 (12)	
H15A	0.7287	0.1410	0.3098	0.112*	
H15B	0.8435	0.0566	0.3388	0.112*	
H15C	0.8295	0.2675	0.3343	0.112*	

### Atomic displacement parameters (Å^2^)

**Table:**

	*U*^11^	*U*^22^	*U*^33^	*U*^12^	*U*^13^	*U*^23^
Ag1	0.05358 (16)	0.04805 (15)	0.02636 (12)	0.01328 (11)	0.00167 (9)	0.01001 (10)
N1	0.0354 (12)	0.0327 (12)	0.0226 (10)	−0.0052 (10)	−0.0003 (9)	0.0008 (9)
N2	0.0343 (12)	0.0324 (12)	0.0246 (11)	0.0031 (10)	−0.0010 (9)	0.0005 (9)
N3	0.0288 (12)	0.0498 (15)	0.0335 (12)	0.0031 (11)	0.0000 (10)	−0.0170 (11)
C1	0.0309 (14)	0.0348 (14)	0.0245 (12)	0.0000 (11)	0.0037 (10)	−0.0022 (10)
C2	0.0296 (13)	0.0307 (13)	0.0227 (12)	−0.0005 (11)	0.0026 (10)	0.0000 (10)
C3	0.0349 (14)	0.0309 (14)	0.0273 (13)	0.0024 (11)	0.0039 (11)	−0.0011 (10)
C4	0.0310 (14)	0.0341 (14)	0.0373 (15)	0.0061 (11)	0.0040 (11)	0.0072 (12)
C5	0.0276 (13)	0.0356 (14)	0.0295 (13)	−0.0035 (11)	−0.0055 (10)	0.0081 (11)
C6	0.0337 (15)	0.0436 (16)	0.0268 (13)	0.0037 (12)	−0.0034 (11)	−0.0095 (11)
C7	0.0325 (14)	0.0291 (13)	0.0221 (12)	0.0020 (11)	0.0005 (10)	−0.0053 (10)
C8	0.0316 (14)	0.0283 (13)	0.0273 (12)	−0.0023 (11)	0.0042 (10)	−0.0036 (10)
C9	0.0292 (14)	0.0393 (16)	0.0347 (14)	0.0019 (12)	0.0005 (11)	−0.0060 (12)
C10	0.0355 (15)	0.0484 (17)	0.0369 (15)	−0.0087 (13)	0.0101 (12)	−0.0023 (13)
C11	0.0451 (16)	0.0419 (16)	0.0246 (13)	−0.0021 (13)	0.0066 (11)	0.0027 (11)
P1	0.0471 (5)	0.0444 (5)	0.0736 (6)	0.0043 (4)	0.0254 (5)	−0.0012 (4)
F1	0.086 (4)	0.064 (3)	0.087 (4)	0.018 (3)	0.007 (3)	0.016 (3)
F2	0.052 (3)	0.092 (5)	0.175 (8)	−0.015 (3)	0.027 (5)	0.043 (5)
F3	0.103 (5)	0.094 (4)	0.119 (5)	0.022 (4)	0.022 (4)	−0.057 (4)
F4	0.115 (10)	0.173 (10)	0.102 (7)	−0.101 (9)	0.007 (6)	−0.010 (6)
F5	0.069 (4)	0.118 (6)	0.110 (6)	0.038 (4)	0.014 (4)	0.051 (5)
F6	0.159 (7)	0.143 (7)	0.065 (4)	0.024 (6)	0.044 (4)	−0.022 (4)
F1'	0.171 (11)	0.056 (5)	0.24 (2)	0.039 (6)	0.097 (12)	0.046 (7)
F2'	0.049 (5)	0.153 (10)	0.123 (8)	0.030 (6)	0.036 (5)	0.007 (7)
F3'	0.183 (14)	0.256 (18)	0.076 (7)	0.038 (13)	−0.048 (8)	−0.028 (8)
F4'	0.083 (9)	0.215 (18)	0.105 (10)	−0.012 (9)	0.063 (8)	0.054 (10)
F5'	0.077 (5)	0.063 (4)	0.129 (7)	0.016 (3)	0.047 (5)	−0.005 (4)
F6'	0.154 (10)	0.152 (10)	0.059 (4)	−0.049 (8)	0.002 (5)	−0.006 (5)
N4	0.084 (3)	0.080 (3)	0.0521 (19)	−0.026 (2)	0.0085 (17)	−0.0043 (17)
C12	0.067 (2)	0.051 (2)	0.0397 (18)	−0.0057 (18)	0.0052 (16)	−0.0009 (15)
C13	0.068 (3)	0.068 (3)	0.088 (3)	−0.002 (2)	0.013 (2)	0.013 (2)
N5	0.067 (2)	0.156 (4)	0.057 (2)	0.044 (3)	0.0217 (18)	0.025 (2)
C14	0.0406 (18)	0.071 (2)	0.060 (2)	0.0125 (17)	0.0159 (16)	0.0091 (19)
C15	0.092 (3)	0.071 (3)	0.074 (3)	−0.001 (2)	0.044 (3)	0.003 (2)

### Geometric parameters (Å, º)

**Table:**

Ag1—N1^i^	2.163 (2)	C9—H9	0.9500
Ag1—N2	2.166 (2)	C10—C11	1.385 (4)
Ag1—Ag1^ii^	3.3023 (5)	C10—H10	0.9500
N1—C1	1.338 (3)	C11—H11	0.9500
N1—C5	1.341 (4)	P1—F3'	1.435 (10)
N1—Ag1^iii^	2.163 (2)	P1—F1'	1.495 (8)
N2—C8	1.342 (3)	P1—F5	1.501 (6)
N2—C9	1.343 (4)	P1—F6	1.512 (5)
N3—C2	1.379 (3)	P1—F4'	1.532 (14)
N3—C6	1.435 (3)	P1—F4	1.539 (12)
N3—H3A	0.84 (3)	P1—F2	1.570 (7)
C1—C2	1.401 (3)	P1—F2'	1.596 (11)
C1—H1	0.9500	P1—F3	1.617 (6)
C2—C3	1.396 (4)	P1—F5'	1.629 (9)
C3—C4	1.387 (4)	P1—F1	1.669 (5)
C3—H3	0.9500	P1—F6'	1.687 (8)
C4—C5	1.380 (4)	N4—C12	1.127 (5)
C4—H4	0.9500	C12—C13	1.452 (6)
C5—H5	0.9500	C13—H13A	0.9800
C6—C7	1.500 (4)	C13—H13B	0.9800
C6—H6A	0.9900	C13—H13C	0.9800
C6—H6B	0.9900	N5—C14	1.110 (5)
C7—C8	1.383 (4)	C14—C15	1.445 (5)
C7—C11	1.387 (4)	C15—H15A	0.9800
C8—H8	0.9500	C15—H15B	0.9800
C9—C10	1.375 (4)	C15—H15C	0.9800
			
N1^i^—Ag1—N2	170.55 (8)	C10—C11—H11	120.5
N1^i^—Ag1—Ag1^ii^	100.46 (6)	C7—C11—H11	120.5
N2—Ag1—Ag1^ii^	84.17 (6)	F3'—P1—F1'	98.8 (9)
C1—N1—C5	119.3 (2)	F5—P1—F6	96.7 (5)
C1—N1—Ag1^iii^	119.39 (18)	F3'—P1—F4'	93.7 (10)
C5—N1—Ag1^iii^	121.31 (17)	F1'—P1—F4'	86.1 (10)
C8—N2—C9	117.9 (2)	F5—P1—F4	90.8 (9)
C8—N2—Ag1	121.18 (18)	F6—P1—F4	92.8 (7)
C9—N2—Ag1	120.91 (17)	F5—P1—F2	94.0 (5)
C2—N3—C6	121.4 (2)	F6—P1—F2	90.7 (5)
C2—N3—H3A	117 (2)	F4—P1—F2	173.7 (8)
C6—N3—H3A	121 (2)	F3'—P1—F2'	96.2 (8)
N1—C1—C2	122.6 (2)	F1'—P1—F2'	92.7 (6)
N1—C1—H1	118.7	F4'—P1—F2'	170.1 (8)
C2—C1—H1	118.7	F5—P1—F3	91.0 (4)
N3—C2—C3	122.6 (2)	F6—P1—F3	172.3 (4)
N3—C2—C1	119.6 (2)	F4—P1—F3	86.5 (6)
C3—C2—C1	117.8 (2)	F2—P1—F3	89.3 (5)
C4—C3—C2	118.8 (2)	F3'—P1—F5'	88.8 (8)
C4—C3—H3	120.6	F1'—P1—F5'	172.5 (8)
C2—C3—H3	120.6	F4'—P1—F5'	93.3 (11)
C5—C4—C3	120.0 (3)	F2'—P1—F5'	86.6 (6)
C5—C4—H4	120.0	F5—P1—F1	173.5 (4)
C3—C4—H4	120.0	F6—P1—F1	89.4 (4)
N1—C5—C4	121.5 (2)	F4—P1—F1	91.3 (9)
N1—C5—H5	119.2	F2—P1—F1	83.5 (4)
C4—C5—H5	119.2	F3—P1—F1	82.9 (3)
N3—C6—C7	111.4 (2)	F3'—P1—F6'	171.5 (8)
N3—C6—H6A	109.3	F1'—P1—F6'	89.8 (8)
C7—C6—H6A	109.3	F4'—P1—F6'	87.3 (8)
N3—C6—H6B	109.3	F2'—P1—F6'	82.9 (5)
C7—C6—H6B	109.3	F5'—P1—F6'	82.7 (5)
H6A—C6—H6B	108.0	N4—C12—C13	179.6 (5)
C8—C7—C11	118.0 (2)	C12—C13—H13A	109.5
C8—C7—C6	120.1 (3)	C12—C13—H13B	109.5
C11—C7—C6	121.9 (2)	H13A—C13—H13B	109.5
N2—C8—C7	123.4 (2)	C12—C13—H13C	109.5
N2—C8—H8	118.3	H13A—C13—H13C	109.5
C7—C8—H8	118.3	H13B—C13—H13C	109.5
N2—C9—C10	122.4 (3)	N5—C14—C15	177.5 (4)
N2—C9—H9	118.8	C14—C15—H15A	109.5
C10—C9—H9	118.8	C14—C15—H15B	109.5
C9—C10—C11	119.3 (3)	H15A—C15—H15B	109.5
C9—C10—H10	120.3	C14—C15—H15C	109.5
C11—C10—H10	120.3	H15A—C15—H15C	109.5
C10—C11—C7	119.0 (3)	H15B—C15—H15C	109.5
			
C5—N1—C1—C2	0.5 (4)	N3—C6—C7—C8	−102.6 (3)
Ag1^iii^—N1—C1—C2	−179.21 (19)	N3—C6—C7—C11	79.0 (3)
C6—N3—C2—C3	−6.3 (4)	C9—N2—C8—C7	−0.1 (4)
C6—N3—C2—C1	176.2 (3)	Ag1—N2—C8—C7	178.53 (19)
N1—C1—C2—N3	176.7 (3)	C11—C7—C8—N2	−0.6 (4)
N1—C1—C2—C3	−0.9 (4)	C6—C7—C8—N2	−179.0 (2)
N3—C2—C3—C4	−176.8 (3)	C8—N2—C9—C10	0.3 (4)
C1—C2—C3—C4	0.7 (4)	Ag1—N2—C9—C10	−178.3 (2)
C2—C3—C4—C5	−0.3 (4)	N2—C9—C10—C11	0.1 (4)
C1—N1—C5—C4	0.0 (4)	C9—C10—C11—C7	−0.8 (4)
Ag1^iii^—N1—C5—C4	179.7 (2)	C8—C7—C11—C10	1.0 (4)
C3—C4—C5—N1	−0.1 (4)	C6—C7—C11—C10	179.4 (3)
C2—N3—C6—C7	174.7 (3)		

Symmetry codes: (i) *x*+1/2, −*y*+1/2, *z*+1/2; (ii) −*x*+1, −*y*+1, −*z*+1; (iii) *x*−1/2, −*y*+1/2, *z*−1/2.

### Hydrogen-bond geometry (Å, º)

**Table:**

*D*—H···*A*	*D*—H	H···*A*	*D*···*A*	*D*—H···*A*
N3—H3*A*···F3	0.84 (3)	2.49 (3)	3.208 (8)	144 (3)
N3—H3*A*···F5	0.84 (3)	2.46 (3)	3.273 (10)	162 (3)
N3—H3*A*···F5′	0.84 (3)	2.18 (3)	2.999 (10)	167 (3)
C8—H8···N4^ii^	0.95	2.63	3.478 (5)	149
C10—H10···F2^iv^	0.95	2.39	3.176 (9)	140
C13—H13*A*···F2′^iv^	0.98	2.54	3.487 (13)	163
C13—H13*B*···F6^i^	0.98	2.53	3.365 (10)	144
C13—H13*B*···F1′^i^	0.98	2.59	3.57 (2)	171
C15—H15*A*···F1	0.98	2.52	3.441 (9)	157
C15—H15*A*···F3′	0.98	2.42	3.356 (13)	160
C15—H15*B*···F2^v^	0.98	2.23	3.088 (10)	146
C15—H15*B*···F5′^v^	0.98	2.57	3.509 (11)	160
C15—H15*C*···F1^iv^	0.98	2.36	3.329 (8)	168
C15—H15*C*···F1′^iv^	0.98	2.09	3.037 (9)	161

Symmetry codes: (i) *x*+1/2, −*y*+1/2, *z*+1/2; (ii) −*x*+1, −*y*+1, −*z*+1; (iv) −*x*+3/2, *y*+1/2, −*z*+1/2; (v) −*x*+3/2, *y*−1/2, −*z*+1/2.
